# Exploring the Traditional Uses of *Thymbra capitata* Infusion in Algarve (Portugal): Anti-Inflammatory, Wound Healing, and Anti-Aging

**DOI:** 10.3390/ph16091202

**Published:** 2023-08-23

**Authors:** Jorge Miguel Alves-Silva, Sónia Pedreiro, Maria Teresa Cruz, Lígia Salgueiro, Artur Figueirinha

**Affiliations:** 1 Univ Coimbra, Institute for Clinical and Biomedical Research, Health Sciences Campus, Azinhaga de S. Comba, 3000-548 Coimbra, Portugal; jmasilva@student.ff.uc.pt; 2 Univ Coimbra, Faculty of Pharmacy, Health Sciences Campus, Azinhaga de S. Comba, 3000-548 Coimbra, Portugal; uc2007119618@student.uc.pt (S.P.); trosete@ff.uc.pt (M.T.C.); amfigueirinha@ff.uc.pt (A.F.); 3 Associated Laboratory for Green Chemistry (LAQV) of the Network of Chemistry and Technology (REQUIMTE), University of Porto, 4099-002 Porto, Portugal; 4 Univ Coimbra, Center for Neuroscience and Cell Biology, Faculty of Medicine, Rua Larga, 3004-504 Coimbra, Portugal; 5 Univ Coimbra, Chemical Process Engineering and Forest Products Research Centre, Department of Chemical Engineering, Faculty of Sciences and Technology, 3030-790 Coimbra, Portugal

**Keywords:** inflammaging, inflammation, oxidative stress, senescence, phenolic composition

## Abstract

Inflammation plays a pivotal role in the resolution of infection or tissue damage. In addition, inflammation is considered a hallmark of aging, which in turn compromises wound healing. *Thymbra capitata* is an aromatic plant, whose infusion is traditionally used as an anti-inflammatory and wound-healing agent. In this study, a *T. capitata* infusion was prepared and characterized by HPLC-PDA-ESI-MSn and its safety profile determined by the resazurin metabolic assay. The anti-inflammatory potential was revealed in lipopolysaccharide (LPS)-stimulated macrophages by assessing nitric oxide (NO) release and levels of inducible nitric oxide synthase (iNOS) and the interleukin-1β pro-form (pro-IL-1β). Wound-healing capacity was determined using the scratch assay. The activity of senescence-associated β-galactosidase was used to unveil the anti-senescent potential, along with the nuclear accumulation of yH2AX and p21 levels. The antiradical potential was assessed by DPPH and ABTS scavenging assays. The infusion contains predominantly rosmarinic acid and salvianolic acids. The extract decreased NO, iNOS, and pro-IL-1β levels. Interestingly, the extract promoted wound healing and decreased β-galactosidase activity, as well as yH2AX and p21 levels. The present work highlights strong antiradical, anti-inflammatory, and wound healing capacities, corroborating the traditional uses ascribed to this plant. We have described, for the first time for this extract, anti-senescent properties.

## 1. Introduction

Inflammation is required for tissue repair upon pathogen invasion or endogenous signals. Indeed, the binding of pathogen-associated molecular patterns (PAMPs) and/or danger-associated molecular patterns (DAMPs), from exogenous or self-origin, respectively, to their receptors, trigger several signaling pathways, such as nuclear factor kappa B (NF-κB), mitogen-activated protein kinase (MAPK), and Janus tyrosine kinase–Signal transducer and activator of transcription (JAK-STAT). Activating these pathways will ultimately lead to the production of several inflammatory markers, such as interleukin-1β, interferon-γ, nitric oxide (NO), and prostaglandins, via inducible nitric oxide synthase (iNOS) and cyclooxygenase-2 activity, respectively [1]. However, several environmental and lifestyle factors can cause the dysregulation of these pathways leading to a sustained inflammatory condition, called low-grade chronic inflammation. This condition is often called “sterile” inflammation since it relies mainly on the activation of inflammatory pathways via DAMPs, released during cellular stress or damage, in the absence of infection. This prolonged state will eventually lead to damage to tissues and organs, which is usually associated with the production of reactive oxygen species (ROS), thus leading to the emergence of several pathologies, such as metabolic syndrome, cardiovascular diseases, and cancer, thus showing increased disability and mortality [2]. One of the main leading causes of this dysregulated inflammatory response is aging. Indeed, this age-related chronic inflammatory state is often called “inflammaging” [3], characterized by elevated levels of circulating pro-inflammatory mediators and a shift towards cellular senescence [3].

Cellular senescence is highly relevant in wound healing, among various other cellular events and in the development of age-related diseases [4]. Indeed, senescent fibroblasts have been shown to be required for optimal wound healing [5]; however, the events needed for a successful healing are often compromised in aging which leads to the accumulation of senescent cells in the tissues [6]. Cellular senescence is triggered by a variety of stimuli, including mitochondrial dysfunction, which leads to the production of ROS and consequently inducing oxidative stress. Oxidative stress is considered as an imbalance between the production of ROS and their scavenging by protective mechanisms, which can lead to chronic inflammation [7], for instance through nucleotide-binding oligomerization domain (NOD)-, leucine-rich repeat (LRR)-, and pyrin domain-containing protein 3 (NRLP3) inflammasome activation [8]. 

Interestingly, it has been shown that blocking NRLP3 [9] or NF-κB [10] signaling pathways retards age-related diseases’ onset. Additionally, it is well-known that senescent cells accumulate in aged tissues and have been shown to play a causal role in age-related pathologies through their proinflammatory secretome, thus strengthening the link between inflammation, cellular senescence, and aging. The relevance of senescence in pathological events has triggered the active exploration of senolytics, aimed at preventing the accumulation of senescent cells during aging, and senomorphics, which target the senescence-associated secretory phenotype, thus reducing the burden of cell senescence [11]. In this context, aromatic and medicinal plants are emerging as a potential source of anti-aging compounds due to their antioxidant and anti-inflammatory properties. Accordingly, several reviews have highlighted the potential of phytochemicals in the management of aging and age-related diseases [12,13]. Although several pathways may be targeted by phytochemicals [13], NF-κB appears at the forefront [14]. Furthermore, the beneficial effects of diet in the prevention of inflammation and inflammaging have recently been highlighted [15], with particular interest in the Mediterranean diet, associated with the consumption of aromatic and medicinal plants. *Thymbra capitata* (L.) Cav. (syn. *Thymus capitatus* (L.) Hoffmans and Link.; *Coridothymus capitatus* (L.) Rchb.f.), commonly known as Spanish oregano and conehead thyme, is an aromatic perennial shrub that grows in xerophilous soils from the Mediterranean basin. In the Iberian Peninsula, it is mainly found in the coastal southern reaches, rarely moving inland [16]. Several traditional uses are ascribed for this species, as presented elsewhere [17], particularly as a wound healing promoter [18], as an anti-inflammatory agent [19,20,21], and against several afflictions of the respiratory system [18,19,20,21]. However, studies validating these therapeutic effects are scarce and the underlying molecular mechanisms have not been properly investigated. Indeed, only a few studies have addressed the anti-inflammatory potential of certain extracts of this species, particularly essential oils, decoctions, and methanolic extracts [22,23,24]. Considering that most of the traditional claims for *T. capitata* recommend the consumption of infusions, this study aims to validate the anti-inflammatory and wound-healing properties of this type of extract. In addition, the chemical composition of the extract was unveiled using high-performance liquid chromatography–photodiode array-electrospray ionization–tandem mass spectrometry (HPLC-PDA-ESI-MS^n^). Envisioning the promotion of the medicinal interest in *T. capitata* and considering the role that aging plays in chronic inflammation and defective wound healing [3,6], we then assessed the anti-senescence effect of the extract, herein reported for the first time for infusions. In addition, considering the role that oxidative stress plays in inflammation, the antiradical potential of the extract will also be reported.

## 2. Results

### 2.1. Phytochemical Characterization

**Figure 1:**
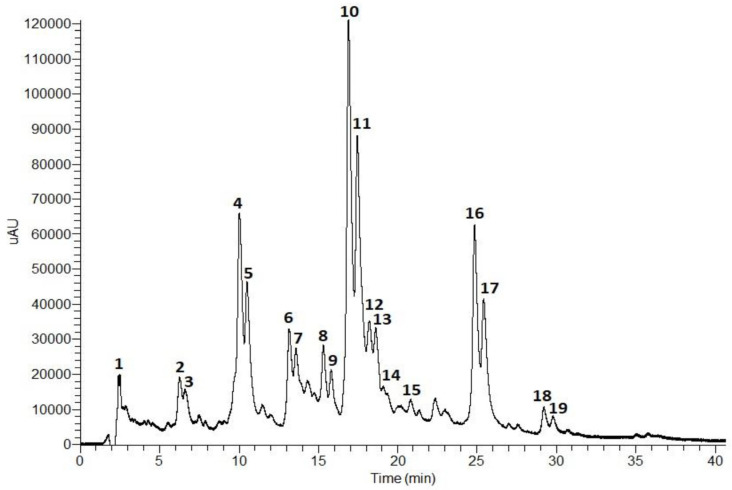
Total scan (230–600 nm) of the HPLC-PDA-ESI-MS^n^ chromatogram (0–40 min) of an *T. capitata* infusion.

The composition of the *T. capitata* infusion was determined by HPLC-PDA-ESI-MS^n^. The results are shown in Figure 1 and Table 1.

According to the phenolic profile, the infusion of *T. capitata* was mainly composed of hydroxycinnamic acids and flavonoids. Hydroxycinnamic acid derivatives comprise the major compounds in the sample, with rosmarinic acid isomers (Peaks **10** and **11**, Figure 1) being the main constituents, followed by salvianolic acids H/I (Peaks **4** and **5**, Figure 1), and salvianolic acid A isomers (Peaks **16** and **17**, Figure 1). Low levels of salvianolic acids B and E were also identified. Among the flavonoids, flavones (Peaks **2** and **3**), flavonols (Peaks **6** and **7**), and flavanones (Peaks **8** and **9**) were found in glycosylated form.

Peak **1** has been tentatively identified as citric acid based on its molecular ion [M − H]^−^ at *m*/*z* 161 and MS^2^ fragments at *m*/*z* 111 (base peak), resulting from dehydration and decarboxylation ([M − H − 2H_2_O − CO_2_]^−^), and at *m*/*z* 173 as a result of dehydration [M − H − H_2_O]^−^ [25,39]. 

The fragmentation patterns of Peaks **4** and **5** were identical to the molecular ion [M − H]^−^ at *m*/*z* 537 and the base peaks at *m*/*z* 339 in MS^2^ and MS^3^ due to the loss of a danshensu moiety [M − H − 198]^−^. Thus, Peaks **4** and **5** were tentatively assigned as salvianolic acid H/I [30,31]. 

The fragmentation pattern of Peaks **10** and **11** showed the molecular ion [M − H]^−^ at *m*/*z* 359 and MS^2^ and MS^3^ base peaks at *m*/*z* 161 corresponding to the release of a danshensu moiety [M − H − 198]^−^. Also, the MS2 fragment at *m*/*z* 179 corresponds to a caffeic acid moiety. In addition, the UV profiles of both peaks are characteristic of hydroxycinnamic acids, namely ferulic or caffeic acid derivatives. Taken together, Peaks **10** and **11** were tentatively identified as rosmarinic acid isomers [32,36,44,45].

Peaks **13**, **14**, **15**, **18**, and **19** presented similar fragmentation patterns with the molecular ion [M − H]^−^ at *m*/*z* 717 and MS^2^ fragments at *m*/*z* 519 due to the loss of a danshensu unit [M − H − 198]^−^. However, Peaks **13** and **14** showed an MS^3^ fragment at *m*/*z* 357 resulting from the loss of two caffeic acids units [M − H − 180 − 180]^−^. Peak **15** presented an MS^3^ fragment at *m*/*z* 321 which originated from the release of another danshensu moiety [M − H − 180 − 180]^−^. Thus, Peaks **13** and **14** were tentatively assigned as salvianolic acid E isomers [31,38,39,40], and Peak **15** as salvianolic acid B [31,40]. Peaks **18** and **19** presented an MS^3^ base peak at *m*/*z* 339 (loss of a caffeic acid unit). Salvianolic acids B, E, L, and isosalvianolic acid B have the same molecular ion and, sometimes, the same MS fragments. However, they can be distinguished by their typical retention times and elution order in reversed phase chromatography [40]: Salvianolic acid E elutes first, then salvianolic acid B, followed by isosalvianolic acid B, and salvianolic acid L [43]. Thus, Peaks **18** and **19** were tentatively identified as isosalvianolic acid B and salvianolic acid L, respectively [38,43]. 

Peaks **16** and **17** were tentatively assigned as salvianolic acid A isomers [41,42]. Both peaks presented the same molecular ion [M − H]^−^ at *m*/*z* 493 and the MS^2^ base peak at *m*/*z* 359 ([M − H − 134]^−^), and MS^3^ base peak at *m*/*z* 161 due to the loss of a danshensu moiety. The occurrence of the MS^3^ fragment at *m*/*z* 179 also suggests the existence of a caffeic acid moiety [41,42]. 

The molecular ion [M − H] ^−^ at *m*/*z* 593 was observed for Peaks **2**, **3**, **6**, and **7**. However, they presented differences in MS^2^ and MS^3^ fragments, and in their UV profiles. Peaks **2** and **3** presented MS^2^ fragments at *m*/*z* 473 (base peak) and 503 that correspond to the loss of 120 a.m.u and 90 a.m.u., respectively, showing a typical fragmentation pattern of di-*C*-glycosylated flavonoids. The MS^3^ base peak at *m*/*z* 353 and fragment at *m*/*z* 383 suggests the presence of another hexosyl unit. Peaks **2** and **3** presented similar UV profiles with absorption maxima at 271 (band II) and 330 nm (band I), characteristic of flavones containing one free hydroxyl group in the B ring [46]. Taken together, the data suggest that Peaks **2** and **3** are apigenin-6,8-di-*C*-hexoside isomers [27,28,29]. Peaks **6** and **7** showed an MS^2^ base peak at *m*/*z* 285 which resulted from the loss of 308 a.m.u. corresponding to a deoxyhexosyl-hexoside unit. An MS^3^ base peak at *m*/*z* 285 indicates the presence of the aglycone (kaempferol) released from the loss of the sugar unit. The UV profile exhibited two bands at 280 and 346 nm, characteristic of flavonols. Thus, Peaks **6** and **7** were tentatively assigned as kaempferol-*O*-deoxyhexosyl-hexoside [32,33]. 

Peaks **8** and **9** exhibited similar UV profiles and MS fragmentation. Concerning their UV profiles, both peaks showed a major band around 284 nm and a small secondary band at 328 nm which is characteristic of flavanones or flavanonols [47]. The molecular ion [M − H] observed at *m*/*z* 609 with MS^2^ and MS^3^ base peaks at *m*/*z* 301 that correspond to the release of a deoxyhexosyl-hexoside moiety [M − H − 308]^−^, characteristic of an O-diglycoside. Peaks **8** and **9** were tentatively identified as an hesperitin-7-*O*-deoxyhexosyl-hexoside isomer [34,35].

Peak **12** exhibited a UV profile typical of flavones. The molecular ion [M − H]^−^ occurs at *m*/*z* 607 with MS^2^ and MS^3^ fragments at *m*/*z* 299 (due to the release of a deoxyhexosyl-hexoside unit) and 284, respectively. Peak **12** was tentatively assigned as diosmetin-*O*-deoxyhexosyl-hexoside [37].

### 2.2. T. capitata Infusion Is Safe towards All Tested Cell Lines

**Figure 2:**
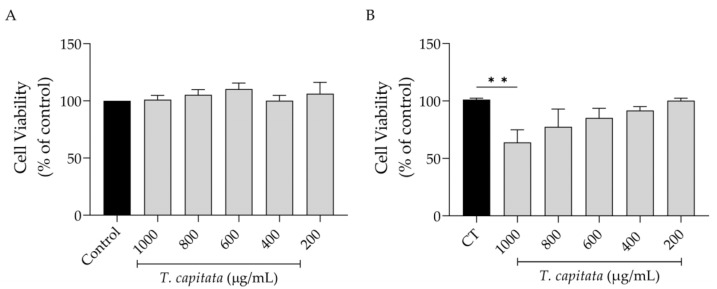
Effect of *T. capitata* extract on RAW 264.7 macrophages (**A**) and NIH/3T3 fibroblasts (**B**). Results show the mean ± standard error of the mean (SEM) of at least three independent experiments. Black bars represent untreated cells. Gray bars represent extract-treated cells. ** *p* < 0.01 after ANOVA followed by Dunnett’s multiple comparison test.

Keeping in mind a possible pharmaceutical application of this extract, we first assessed its effect on cell viability. As shown in Figure 2, the extract was completely safe for macrophages (Figure 2A) as observed by control-like values in all tested concentrations. However, in fibroblasts, a significant decrease in viability was detected at the highest dose tested (1000 µg/mL, Figure 2B).

### 2.3. T. capitata Extract Presents Strong Anti-Inflammatory Potential in LPS-Stimulated Macrophages

**Figure 3:**
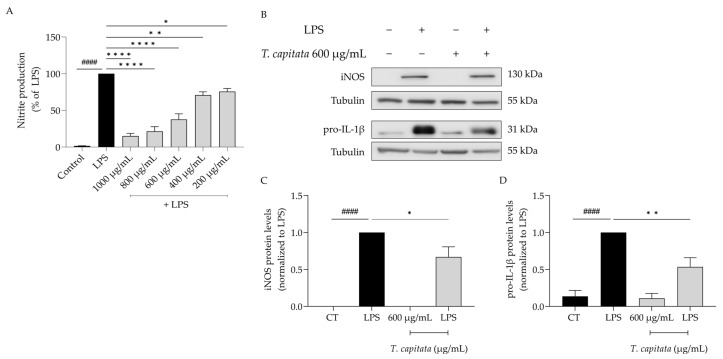
*T. capitata* extract exerts anti-inflammatory effects. Effect on nitrite release (**A**). Representative Western blots (**B**) and relative protein levels of iNOS (**C**) and pro-IL-1β (**D**). Black bars represent untreated cells and grey bars represent *T. capitata*-treated cells. Results show the mean ± standard error of the mean (SEM) of at least three independent experiments. #### *p* < 0.0001 when compared to CT, * *p* < 0.05, ** *p* < 0.01 and, **** *p* < 0.0001 when compared to LPS after ANOVA followed by Tukey’s multiple comparison test.

Considering the anti-inflammatory uses ascribed to infusions of *T. capitata*, we aimed to validate those effects on lipopolysaccharide (LPS)-stimulated macrophages. As expected, the presence of the Toll-like-4 agonist LPS for 24 h induced the release of nitric oxide, quantified as nitrites ([NO_2_^−^] = 43.66 ± 4.05 µM). The addition of the extract was able to decrease the amount of NO in the supernatant in all tested doses (IC_50_ = 558.6 µg/mL, Figure 3A). To unveil the mechanism of action associated with the anti-inflammatory effects of the extract, the dose of 600 µg/mL was selected, as it was the lowest dose with a more than 50% reduction in NO release. As shown in Figure 3B–D, the presence of LPS alone greatly increased the protein levels of both iNOS and the IL-1β pro-form. Interestingly, when *T. capitata* was added, the expression of both proteins decreased, suggesting that the infusion might modulate the NF-κB signaling pathway, a pro-inflammatory transcription factor controlling the expression of both mediators. 

### 2.4. T. capitata Extract Promotes Wound Healing

**Figure 4:**
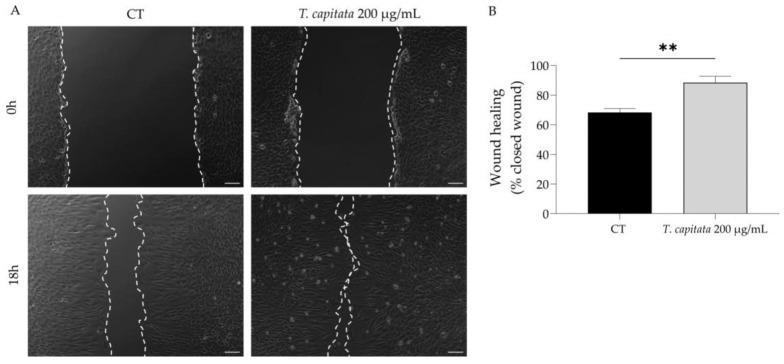
Effect of *T. capitata* extract on the wound healing of NIH/3T3 fibroblasts. Representative bright-field images of NIH/3T3 fibroblasts 0 h and 18 h (**A**) and percentage of closed wound (**B**) of NIH/3T3 fibroblasts 18h after scratch wound assay, in the presence (gray bar) or absence (black bar) of the extract. Results show the mean ± standard error of the mean (SEM) of at least three independent experiments. ** *p* < 0.01 when compared to CT after unpaired Student’s *t*-test. Scale bar: 100 µm.

To shed light on the wound healing promotion ascribed to the infusion of *T. capitata*, we used the scratch wound assay to assess the effect of the extract on wound healing. As observed in Figure 4, the infusion significantly promoted wound healing, thus sustaining the claims ascribed to this plant. 

### 2.5. Antioxidant Potential

**Figure 5:**
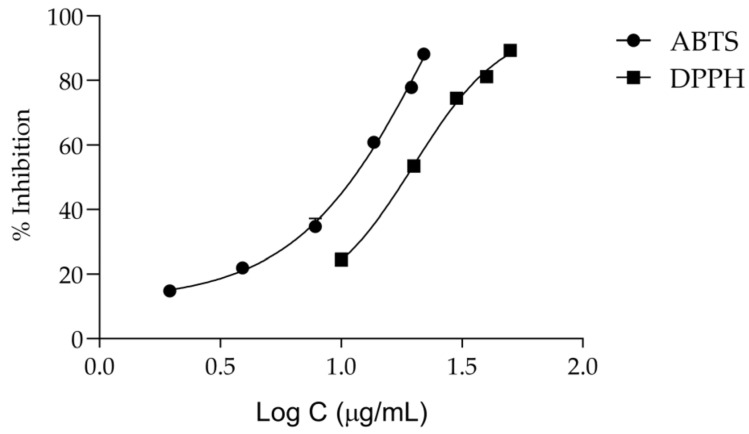
Dose–response curves for the anti-scavenging activity of the *Thymbra capitata* infusion in DPPH and ABTS assays. Each result represents the mean ± SD of three independent assays, performed in duplicate.

Keeping in mind that oxidative stress plays a central role in the onset of inflammation and aging [7], we then assessed the antiradical activity of the extract. According to Figure 5 and Table 2, *T. capitata* extracts have a considerable antiradical activity, displaying an IC_50_ of 19.85 ± 1.08 μg/mL for the DPPH assay and 11.53 ± 0.27 μg/mL for the ABTS assay.

### 2.6. T. capitata Extract Exerts Anti-Senescence Effects

**Figure 6:**
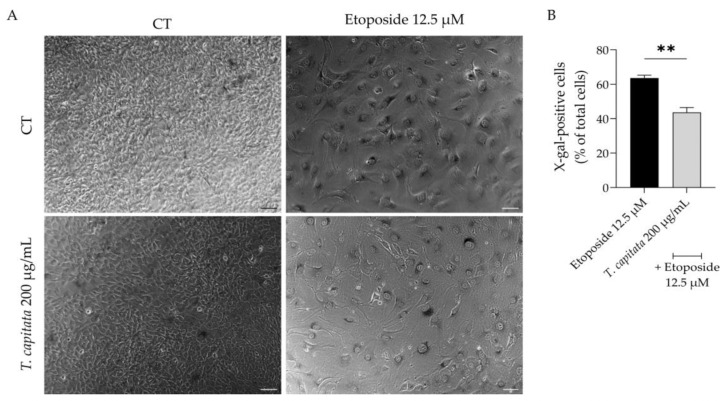
Effect of *T. capitata* extract on senescence-associated β-galactosidase activity. Representative bright-field images (**A**) and percentage of X-galactose-positive cells (**B**) of NIH/3T3 fibroblasts treated with etoposide (12.5 µM) for 24 h, and then left in culture medium alone (black bar) or in the presence of *T. capitata* extract (gray bar) for 72 h. Results show the mean ± standard error of the mean (SEM) of at least three independent experiments. ** *p* < 0.01 when compared to etoposide after an unpaired Student’s *t*-test. Scale bar: 100 µm.

Considering that aging contributes to low-grade chronic inflammation and compromises wound healing [3,6], we then assessed whether the extract was able to alleviate cell senescence. Senescent cells have several morphological and biochemical characteristics that are used for their detection in vitro and/or in vivo. No single marker is sufficient to unequivocally identify a senescent cell; thus, a combination of markers was herein used to increase the specificity of detection and to prove the anti-senescence effect of the extract. Senescence was induced with etoposide (12.5 µM) for 24 h, which as expected led to a huge increase in the activity of β-galactosidase activity (Figure 6A,B), a sign of senescent cells, when cells were left to recover in etoposide-free medium for 72 h. Interestingly, when the extract was added in the recovery phase, the percentage of β-galactosidase-positive cells was reduced (Figure 6A,B), thus suggesting anti-senescent effects for the extract. 

**Figure 7:**
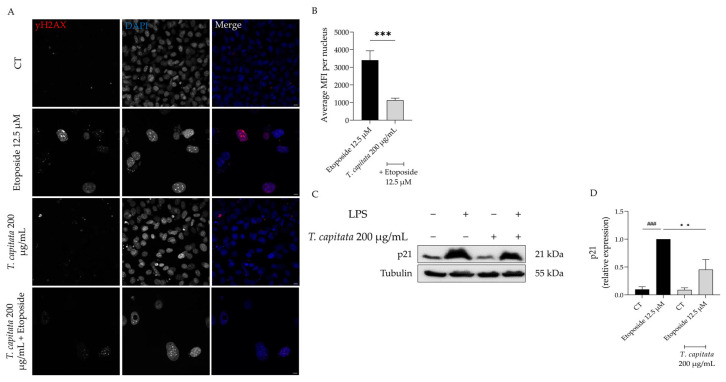
Effect of *T. capitata* extract on yH2AX (**A**,**B**) and p21 protein levels (**C**,**D**). Representative Z-stack images of NIH/3T3 fibroblasts treated with Etoposide (12.5 µM) for 24 h, and then left in culture medium alone (black bar) or in the presence of *T. capitata* extract (gray bar) for 72 h. yH2AX was stained with Alexa Fluor 564 and nuclei were counterstained with DAPI (**A**). Quantification of the yH2AX average mean fluorescence intensity (MFI) per nucleus (**B**). A minimum of 10 images were quantified in three independent experiments. *** *p* < 0.001 when compared to etoposide after a Mann–Whitney test. Scale bar: 10 µm. Representative Western blot images (**C**) and relative protein expression of p21 (**D**). Results show the mean ± standard error of the mean (SEM) of at least three independent experiments. ### *p* < 0.001 when compared to CT, ** *p* < 0.01 when compared to etoposide after ANOVA followed by Dunnett’s multiple comparison test.

In order to further corroborate these results, we then assessed the accumulation of the phosphorylated form of the H2AX histone (yH2AX) in the nucleus, as well as the expression of p21, a key player in senescence [48]. As observed in Figure 7A,B, an increase in the mean fluorescence intensity was observed in etoposide-treated cells, whereas in the presence of the extract this value was significantly reduced. Similarly, the protein levels of p21 were significantly decreased in cells treated with the extract when compared to the etoposide-only cells (Figure 7C,D). Taking these results, we hypothesized that *T. capitata* exerts anti-senescent effects by modulating the p53/p21 signaling pathway.

## 3. Discussion

Considering the increase in life expectancy, which has increased from 66.8 years in 2000 to 73.3 years in 2019 according to the World Health Organization [49], and the link between inflammation, aging, and wound healing [3,6], there is increased interest in the research of senotherapeutics for the management of age-related diseases [50]. Furthermore, senotherapeutic agents that present other relevant biological properties are of special interest. In this context, aromatic and medicinal plants appear at the forefront due to their reported traditional uses. Indeed, based on the traditional uses ascribed to *T. capitata*, the present study was designed in order to shed some light on its uses, as well as to further promote its medicinal interest by ascribing other relevant properties. Herein, we report for the first time for an infusion: anti-inflammatory effects, probably by modulation of the NF-κB signaling pathway; and wound healing properties, thus, bringing scientific evidence to the empirical and traditional uses of *T. capitata*. Furthermore, we also report that this extract possesses antiradical properties, as well as anti-senescence effects by modulation of the p21/p53 pathway.

Our chemical analysis of the extract demonstrated that rosmarinic acid and salvianolic acids are the predominant compounds, with the presence of apigenin glycosides (apigenin-6,8-di-*C*-hexoside) and flavonol derivatives (kaempferol-*O*-deoxyhexosyl-hexoside) also reported. Our results are in agreement with previous works using this species. Indeed, Llorent-Martínez and colleagues [23] studied the phenolic profile of infusions from *T. capitata* and *Thymus sipyleus* subsp. *rosulans* by HPLC-ESI-MS^n^, having identified rosmarinic acid as the major polyphenol. The authors also identified salvianolic acids A, B, and E, and apigenin-6,8-di-*C*-glucoside [23]. Using a methanolic extract from Tunisian *T. capitata*, Jaouadi and colleagues also identified rosmarinic acid as the main polyphenol, followed by salvianolic acids A and E and apigenin-*C*-di-hexoside [35]. Interestingly, our study also reports the presence of salvianolic acids H, I, and L, as well as isosalvianolic acid B being the first to detect such compounds in *T. capitata* and in other species of the genus *Thymbra*.

Here, we report that the infusion of *T. capitata* is able to decrease NO release in LPS-stimulated macrophages by decreasing the protein levels of iNOS, probably by inhibiting the NF-κB signaling pathway, since a decrease in pro-IL-1β, whose expression is also controlled by NF-κB [51], was also reported. The anti-inflammatory potential of non-volatile extracts of *T. capitata* has been scarcely reported. The decoction water of *T. capitata* was reported to inhibit 5-lypoxygenase activity [22]. A study compared the activity of a methanolic extract and infusion from *T. capitata* and demonstrated that both extracts decreased the gene expression of *cyclooxygenase*-*2* and *interleukin 6*, with the methanolic extract being more active [23]. Regarding isolated compounds, several studies have been conducted particularly for rosmarinic and salvianolic acids. Indeed, rosmarinic acid is reported to exerts its anti-inflammatory properties alone [52,53,54,55,56,57,58,59,60] or encapsulated in nanovesicles [61] or associated with chitosan [62,63]. The ability to modulate NRLP3 inflammasomes [56,61], as well as SIRT1/NF-κB [58] and TLR-4/NF-κB/STAT3 [57] pathways, has been associated with rosmarinic acid. Similarly, the inhibition of NF-κB activation is also reported for salvianolic acid A [64,65]. Apigenin-6,8-di-*C*-hexoside, one of the most common flavonoids of the studied extract, significantly inhibited the inflammation in a carrageenan-induced rat hind paw edema model. Moreover, the authors studied the anti-inflammatory activity of this compound in peritoneal macrophages and showed that it was able to decrease NO and TNF-α levels, and it prevented NF-κB nuclear translocation [66]. Having in mind the reported effects for the isolated compounds and their capacity to modulate the NF-κB pathway, we suggest that the activity reported for the extract might be attributed to their presence. 

Strikingly, although a *T. capitata* infusion is traditionally used as a wound healing agent, our study is the first to scientifically validate those claims. Indeed, we reported that the extract is able to greatly promote the migration of fibroblasts in the scratch wound assay. Regarding isolated compounds, several studies have reported their wound healing capacities. Indeed, wound healing properties have been reported for rosmarinic acid [67,68], salvianolic acid A, and danshensu [69,70,71,72]. The aglycone apigenin also promotes in vitro and in vivo wound healing [73,74,75,76,77]. Considering that these compounds are the major phenolic compounds in our extract, we hypothesize that the reported activity might be attributed to their presence; however, the contribution of other minor compounds cannot be discarded.

Herein, we report that a *T. capitata* extract possesses strong antiradical properties as observed by its capacity to scavenge both ABTS and DPPH free radicals. Considering that antioxidant activity is often correlated with the amount of polyphenols [23,78], we suggest that the reported activity might be attributed to the phenolic acids and flavonoids present in the mixture. Indeed, it has been reported that rosmarinic acid inhibits NO release [79], reduces lipid peroxidation [80], and decreases the levels of glutathione (GSH) and malondialdehyde (MDA) [81,82,83,84,85]. This phenolic acid also activates the enzymes catalase (CAT) and glutathione peroxidase (GSH-Px) [81,82,83,84,85]. Salvianolic acids have also been associated with strong antioxidant effects. Indeed, salvianolic acid A was reported as a potent antioxidant compound by FRAP, DPPH, and superoxide anion scavenging assays [86]. Salvianolic acid L exhibits a strong anti-scavenging activity of DPPH and the superoxide anion, being more effective than rosmarinic acid, caffeic acid, and Trolox [87]. The aglycone apigenin decreases ROS levels through a reduction in lipid peroxidation and through membrane protein damage [88]. Another study compared the xanthine oxidase inhibition capacity of apigenin and apigenin 6-*C*-glucoside-8-*C*-arabinose and demonstrated that the glycosylated derivatives are more potent than the aglycone [89]. Kaempferol, kaempferol-7-*O*-rutinoside, kaempferol-7-*O*-rhamnoside, and kaempferol-7-*O*-glucoside demonstrated strong inhibition effects on NO production [90]. Regarding salvianolic acids H and I and isosalvianolic acid B, no studies have been conducted for the best of the authors’ knowledge. Considering the plethora of studies showing strong antioxidant properties for the major compounds of the present extract, we suggest that the reported scavenging potential might be associated with their presence; however, the contribution of other compounds, such as salvianolic acids H and I, cannot be ruled out.

Herein, we report for the first time that an infusion from *T. capitata* exerts strong anti-senescence effects—probably via modulation of the p21/p53 signaling pathway. This assumption is validated by the decrease in yH2AX, as it has been reported that phosphorylation of H2AX (yH2AX) is required for increasing p21 levels and therefore cells enter cell cycle arrest [91]. Previous studies from our group demonstrated anti-senescent properties for the essential oil and hydrodistillation residual water of *T. capitata* [24]; however, studies addressing the mechanisms of action underlying these effects are lacking. Certain studies have reported anti-aging/senescent effects for some of the major compounds found in the extract. Indeed, rosmarinic acid increases lifespan in *C. elegans* [81] and in an animal model of familial amyotrophic lateral sclerosis [92]. Furthermore, it has protective effects on UV-induced aging [93,94] and in other models of cell senescence [84,95,96,97]. The aglycone apigenin [98,99,100,101,102,103,104] also exerts anti-aging effects. With these results in mind, we hypothesize that rosmarinic acid and apigenin might be the major contributors for the reported anti-senescent effects; however, synergistic effects and the contribution of other compounds cannot be discarded.

## 4. Materials and Methods

### 4.1. Plant Material and Sample Preparation

Aerial parts from *Thymbra capitata* L. (*T. capitata*) were collected in 2021 at the flowering stage (May) in Carvoeiro (Algarve, Portugal). The collected parts were air-dried at room temperature (approximately one month) before sample preparation. Voucher specimens were included in the Herbarium of the Faculty of Pharmacy of University of Coimbra, with the accession number Salgueiro 135. After drying, the aerial parts of *T. capitata* were ground (knife mill KSM2, BRAUN, Frankfurt, Germany) and sieved through a 60-mesh sieve. The powdered plant was stored at −20 °C in the dark. Subsequently, an infusion was prepared by adding 5 g of the powdered aerial parts of *T. capitata* to 500 mL of water at 100 °C for 30 min. The infusion was then filtered under vacuum, concentrated in a rotavapor at 40 °C, and frozen for prior lyophilization. After lyophilization, it was stored at −20 °C in the dark. The yield of dry plant was 29.5% (*w*/*w*).

### 4.2. HPLC-PDA-ESI-MS^n^

The phenolic profile of the *Thymbra capitata* infusion was achieved by high-performance liquid chromatography (Finnigan Surveyor, THERMO, Waltham, MA, USA) coupled to a photodiode array (PDA) (Finnigan Surveyor, THERMO) and a linear ion trap mass spectrometer (LIT-MS) (LTQ XL, Thermo Scientific, Waltham, MA, USA). The sample was eluted with the mobile phases of 2% (*v*/*v*) aqueous formic acid (solvent A) and acetonitrile (solvent B) through a Waters Spherisorb ODS2 C18 column (150 × 2.1 mm and 3 μm particle size) (Waters Corp., Milford, MA, USA). The gradient was 5–50% (*v*/*v*) solvent B for 60 min at a flow rate of 200 μL/min, at 20 °C, and the absorption profile of the compounds was recorded at 280 and 320 nm. MS spectra were acquired by negative electrospray ionization (ESI) mass spectrometry. Helium was used as the collision gas with a collision energy of 35%. Nitrogen was used as the nebulizing gas with a sheath gas flow of 35 (arbitrary units) and as an auxiliary gas with a flow of 20 (arbitrary units). The temperature and voltage of the capillary were 275 °C and −35.00 V, respectively. The source voltage was 5.00 kV. For the analysis, a concentration of 500 μg/mL of *T. capitata* infusion was injected. 

### 4.3. Cell Culture

The cell lines RAW 264.7 (mouse leukemic macrophage cell line) and NIH/3T3 (mouse embryonic fibroblast) were obtained from the American Type Culture Collection (ATCC TIB-71 and ATCC CRL-1658, respectively) and were cultured as previously described by the team [105]. Briefly, RAW cells were cultured on endotoxin-free Dulbecco’s Modified Eagle Medium (DMEM) supplemented with 10% (*v*/*v*) non-inactivated fetal bovine serum (FBS), 3.02 g/L sodium bicarbonate, 100 µg/mL streptomycin, and 100 U/mL penicillin at 37 °C in a humidified atmosphere of 95% air and 5% CO_2_. NIH/3T3 were cultured in DMEM with 25 mM glucose, 3.7 g/L of sodium bicarbonate, 100 U/mL of penicillin, and 100 μg/mL of streptomycin supplemented with 10% heat inactivated FBS. Both cell lines were sub-divided when they reached 70–80% confluency. Cell morphology was controlled using an inverted light microscope.

### 4.4. Cell Viability

The effect of different concentrations of the extract on macrophages’ and fibroblasts’ viability was evaluated through the resazurin reduction test, as previously reported [106]. Briefly, macrophages (0.6 × 10^6^ cells/mL) or fibroblasts (1.25 × 10^5^ cells/mL) were seeded in 48-well plates. After an overnight stabilization, 1000–200 µg/mL of the extract prepared in medium was added for 24 h. At the end of the experiment, the medium was removed and fresh medium containing resazurin (1:10) was added for 1 h for macrophages or 2 h for fibroblasts. The absorbance at 570 nm with a reference filter of 620 nm was registered in an automated plate reader (SLT, Salzburg, Austria). Cell viability was determined using the following equation:

Cell viability (%) = (Abs_Exp_/Abs_CT_) × 100

where Abs_Exp_ is the absorbance (difference between 570 and 620 nm) in the different experimental conditions and Abs_CT_ is the absorbance in control cells (no extract).

### 4.5. Anti-Inflammatory Potential

#### 4.5.1. Nitric Oxide Production

The capacity of the extract to decrease the nitric oxide production in lipopolysaccharide (LPS)-stimulated macrophages was assessed using the methodology described by our group [107,108]. Briefly, NO production was measured by quantifying the accumulation of nitrites in culture supernatants, using the Griess reagent [109]. Cells (0.6 × 10^6^ cells/well) were cultured in 48-well culture plates. After an overnight stabilization, macrophages were pre-treated for 1 h with 1000–200 µg/mL of the extract and then activated with 50 ng/mL of LPS during 24 h. Positive (LPS-stimulated macrophages) and negative controls (untreated macrophages) were performed. After this period of incubation, equal volumes of culture supernatants and Griess reagent [1:1 of 0.1% (*w*/*v*) N-(1-naphthyl) ethylenediaminedihydrochloride and 1% (*w*/*v*) sulphanilamide containing 5% (*w*/*v*) H_3_PO_4_] were mixed and incubated for 30 min, in the dark. The absorbance at 550 nm was registered in an automated plate reader (SLT, Salzburg, Austria) and the nitrite concentration was determined from a sodium nitrite standard curve. 

#### 4.5.2. Western Blot Analysis of Pro-Inflammatory Mediators

RAW 264.7 cells (1.2 × 10^6^ cells/well) were cultured in 6-well plates and stabilized overnight. Cells were then subjected to 1 h incubation with the extract (600 µg/mL), followed by 24 h of LPS activation (50 ng/mL). Negative and positive controls comprising untreated and LPS-treated cells, respectively, were included. Cell lysate preparation follow the protocol previously performed in Zuzarte et al. [110].

The protein levels of the inducible nitric oxide synthase (iNOS) and IL-1β pro-form (pro-IL-1β) were assessed by Western blot, as previously described [24]. For protein separation, an electrophoretic assay was performed with 10% (*v*/*v*) SDS-polyacrylamide gels at 130 V during 1.5 h, at room temperature. Protein lines were consequently blotted, during 3 h at 400 mA in a refrigerated (4 °C) wet transference system, to membranes of polyvinylidene fluoride, which were previously activated, at room temperature, with methanol. The membranes were then incubated for 1 h at room temperature with 5% (*w*/*v*) skim milk in TBS-T (Tris Buffered Saline, 50 mM Tris-HCl, pH 7.5, 150 mM NaCl, 2.5 mM KCl, with 0.1% Tween 20). They were further incubated overnight at 4 °C with specific anti-iNOS (1:500; MAB9502, R & D Systems, Minneapolis, MN, USA) or anti-pro-IL-1β (1:1000; ab9722, Abcam, Cambridge, UK) antibodies. Finally, they were washed for 30 min with TBS-T (10 min, 3 times) and incubated for 1 h at room temperature with secondary antibodies (1:40,000; Santa Cruz Biotechnology, Dallas, TX, USA) conjugated with horseradish peroxidase. The immunocomplexes’ detection was performed by a chemiluminescence scanner (Image Quant LAS 500, GE, Boston, MA, USA). An antibody against tubulin (1:20,000; Sigma, Burlington, MA, USA) was used as a loading control. ImageLab software version 6.1.0 (Bio-Rad Laboratories Inc., Hercules, CA, USA) was used for protein quantification.

### 4.6. Cell Migration

The effect of the extract on cell migration was investigated through the scratch wound assay according to Martinotti et al., [111] with slight modifications, as previously reported [24]. Briefly, 3T3 fibroblasts were seeded at 2.5 × 10^5^ cells/mL in 12-well plates. After 24 h of growth, a scratch was made in the cell monolayer using a pipette tip. Detached cells were removed by washing cells with sterile PBS 1×. DMEM with 2% FBS was added to all plates, in the presence or absence of 200 µg/mL of extract prepared in culture medium. Using a phase-contrast microscope, images were acquired 0 and 18 h post-scratch, and the open area was quantified using an ImageJ/FIJI plugin [112]. The results presented were obtained using the following equation:

$$wound\;closure\left(\%\right)=\frac{A_{t=0h}-A_{t=18h}}{A_{t=0h}}\times 100$$

where A_t=0h_ is the area of the wound 0 h after the scratch and A_t=18h_ is the area 18 h post-scratch. 

### 4.7. Antioxidant Assays

#### 4.7.1. DPPH Free Radical Scavenging Assay

The 2,2-diphenyl-1-picrylhydrazyl radical scavenging assay (DPPH) was used to evaluate the antioxidant activity of the extract from *T. capitata*, as described by Pedreiro et al. [113]. For the assay, several dilutions were prepared, and 10 µL of each was added to a reaction medium with 140 µL of methanol, 50 µL of DPPH solution (500 µM in methanol), and 100 µL of acetate buffer (100 mM; pH 6.0). The decrease in absorbance was monitored at 517 nm in microplate reader photometer (Multiskan FC, Thermo Scientific, Waltham, MA, USA) at 30 min. All measurements were performed in duplicate in three independent experiments. DPPH inhibition (%) was calculated from the following formula:

(1) $$DPPH\;inhibition\left(\%\right)=(100-\frac{Absorbance\;of\;DPPH-Absorbance\;of\;sample}{Absorbance\;of\;DPPH})\times 100$$

IC_50_ values were calculated from the plot of % DPPH inhibition against the logarithmic scale of the concentration (log C; µg/mL) using GraphPad Prism version 9.3.0 (GraphPad Software, San Diego, CA, USA). The results are also expressed as TE (Trolox-equivalents).

#### 4.7.2. ABTS Assay

The 2,20-Azinobis-(3-ethylbenzothiazoline-6-sulfonate) (ABTS) assay was used to evaluate the antiradical activity of the extract, as described by Pedreiro et al. (2023) [113]. The ABTS radical was generated by an aqueous solution of 7 mM ABTS●+ and 2.45 mM potassium persulfate (Merck, Darmstadt, Germany). The solution was stored in the dark at room temperature. After 16 h, this solution was adjusted with phosphate-buffered saline (PBS) at pH 7 until an absorbance of 0.7 ± 0.02 at 734 nm was reached. The ABTS assay was carried out by the addition of 50 µL of the extract to 2 mL of ABTS ●+ solution, which was stirred for 10 s and incubated at room temperature in the dark. The extinction of the reaction mixture was measured at 734 nm at 4 min, and the IC_50_ value calculated by interpolating the plot of % of ABTS vs. log C in µg/mL using GraphPad Prism version 9.3.0. Trolox was used as a positive control, and the results were also expressed as TE. All measurements were performed in duplicate in three independent experiments.

### 4.8. Anti-Senescence Potential

#### 4.8.1. Senescence-Associated β-Galactosidase Activity

Senescence was evaluated using the senescence-inducer etoposide, as reported elsewhere [114], with some modifications. Briefly, after 24 h of fibroblasts’ culture in the presence of etoposide, the cells were further incubated for 72 h, in the presence or absence of the extract (200 µg/mL). Beta-galactosidase was assessed using a commercially available kit according to the manufacturer’s protocol (#9860, Cell Signaling Technology Inc., Danvers, MA, USA). The distinct blue color staining indicates beta-galactosidase activity. After color development, the wells were photographed for subsequent image analysis. ImageJ software (version 1.53t) was used for the quantitative analysis, by assessing the percentage of senescent cells.

#### 4.8.2. yH2AX Staining

For the assessment of the nuclear staining of histone yH2AX, NIH/3T3 fibroblasts were seeded at 1 × 10^5^ cells/mL in glass coverslips and treated as reported in Section 4.8.1. At the end of the treatment, cells were fixated with 4% paraformaldehyde for 15 min. Then, after washes with sterile PBS (three times), cells were permeabilized with 0.1% Triton X-100 for 15 min, followed by three washes with PBS. Cells were then blocked with blocking solution (3% bovine serum albumin, 10% goat serum in PBS) for 1 h. Then, primary antibody against yH2AX (1:500, Cell Signaling 9718) prepared in blocking solution was added and the cells incubated overnight at 4 °C. At the end of the incubation period, coverslips were washed with PBS (three times), and incubated for 1 h at room temperature with the corresponding secondary antibody (1:500, goat anti-rabbit Alexa Fluor 564) and DAPI (1:2000 prepared in blocking solution). After washes with PBS, coverslips were mounted in glass slides with Mowiol mounting medium. Images were acquired in a confocal point-scanning microscope (Zeiss LSM710; Carl Zeiss, Oberkochen, Germany) in 40× objective.

#### 4.8.3. p21 Protein Levels

NIH/3T3 fibroblasts (2.5 × 10^5^ cells/mL) were plated in 6-well plates and then treated as reported in Section 4.8.1. Afterwards, cell lysates were prepared as reported by Zuzarte et al. [110]. For protein separation, samples (35 µg/lane) were electrophoretically separated in a 15% (*v*/*v*) SDS–polyacrylamide gel at 130 V, during 1.5 h, at room temperature. Protein lines were consequently blotted, during 3 h at 400 mA, using a refrigerated (4 °C) wet transference system, to membranes of polyvinylidene fluoride, which were previously activated with methanol, at room temperature. The membranes were then incubated for 1 h at room temperature with 5% (*w*/*v*) skim milk in TBS-T. They were further incubated overnight at 4 °C with a specific p21 antibody (1:500, Abcam ab188224). Finally, they were washed for 30 min with TBS-T (10 min, 3 times) and incubated for 1 h at room temperature with secondary antibodies (1:40,000; Santa Cruz Biotechnology) conjugated with horseradish peroxidase. The immunocomplexes’ detection was performed by a chemiluminescence scanner (Image Quant LAS 500, GE, Boston, MA, USA). An antibody against tubulin (1:20,000; Sigma) was used as a loading control. ImageLab software version 6.1.0 (Bio-Rad Laboratories Inc., Hercules, CA, USA) was used for protein quantification.

### 4.9. Statistical Analysis

The experiments were performed at least in duplicate for three independent experiments. Mean values ± SEM (standard error of the mean) are presented in the results. Statistical significance was evaluated by a one-way analysis of variance (ANOVA), unpaired *t*-test, or Mann–Whitney test followed by the appropriate post-hoc test analysis using GraphPad Prism version 9.3.0. *p* values < 0.05 were accepted as statistically significant. 

## 5. Conclusions

**Figure 8:**
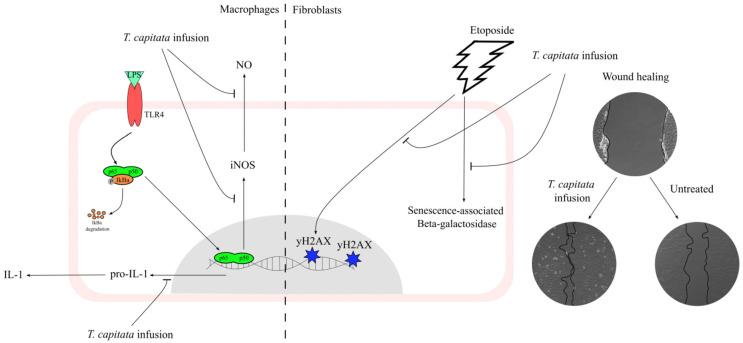
A *T. capitata* infusion exerts anti-inflammatory effects on macrophages by decreasing NO release, due to the inhibition of iNOS and pro-IL-1β protein levels. The extract of *T. capitata* is able to prevent the activity of senescence-associated β-galactosidase and the phosphorylation of histone H2AX. *T. capitata* is able to promote cell migration when compared to untreated cells.

This work contributes to filling the gap in the scientific validation of the traditional uses ascribed to *T. capitata*, particularly those related to inflammation and wound healing, by elucidating the mechanisms underlying those effects as summarized in Figure 8. Indeed, we herein report that the infusion exerts anti-inflammatory effects, probably by inhibiting the NF-κB signaling pathway. Furthermore, we also report that this extract displays wound-healing properties. Considering the pivotal role of oxidative stress and aging on the reported activities, we also demonstrate that the extract has strong antiradical properties, and, for the first time for this type of extract, we demonstrate potent anti-senescence effects.

Overall, the present work describes several activities that are highly relevant to the traditional uses claimed for *T. capitata*, while simultaneously expanding the therapeutic potential of this species to other areas of biomedicine, such as inflammaging.

## Figures and Tables

**Table 1:** Compounds identified in the *T. capitata* infusion by HPLC-PDA-ESI-MS^n^.

Peak	Partial Identification	R_t_ (min.)	λ_max._ by HPLC/PDA (nm)	[M − H]^−^	MS^2^	MS^3^	Ref.
**1**	Citric acid	2.50	237, 250 max, 275 sh	191 (100)	[191]: 173 (35), 111 (100)	-	[25,26]
**2**	Apigenin-6,8-di-*C*-hexoside	6.26	235, 271, 329 max	593 (100)	[593]: 503 (30), 473 (100), 353 (20)	[593 473]: 473 (20), 383 (12), 353 (100)	[27,28,29]
**3**	Apigenin-6,8-di-*C*-hexoside	6.62	236, 271, 330 max	593 (100)	[593]: 593 (30), 503 (35), 473 (100), 383 (20), 353 (25)	[593 473]: 473 (10), 383 (20), 353 (100)	[27,28,29]
**4**	Salvianolic acid H/I	10.13	254, 285, 310 sh, 343 max	537 (100)	[537]: 493 (5), 357 (2), 339 (100)	[357 339]: 339 (100), 295 (80), 228 (60)	[30,31]
**5**	Salvianolic acid H/I	10.49	254, 285, 310 sh, 343 max	537 (100)	[537]: 493 (15), 339 (100)	[357 339]: 339 (100), 295 (50), 229 (70)	[30,31]
**6**	Kaempferol-*O*-deoxyhexosyl-hexoside	13.14	253, 287 sh, 346 max	593 (100)	[593]: 593 (30), 285 (100)	[593 285]: 285 (100)	[32,33]
**7**	Kaempferol-*O*-deoxyhexosyl-hexoside	13.56	253, 287 sh, 345 max	593 (100)	[593]: 593 (35), 285 (100)	[593 285]: 285 (100)	[32,33]
**8**	Hesperitin-7-*O*-deoxyhexosyl-hexoside	15.25	243, 284 max, 328 sh	609 (100)	[609]: 301 (100)	[609 301]: 301 (100), 286 (40), 242 (20)	[34,35]
**9**	Hesperitin-7-*O*-deoxyhexosyl-hexoside	15.79	243, 284 max, 327 sh	609 (100)	[609]: 301 (100)	[609 301]: 301 (100), 286 (35), 242 (30)	[34,35]
**10**	Rosmarinic acid isomer	16.89	251, 291, 329 max	359 (100)	[359]: 223 (30), 197 (35), 179 (35), 161 (100), 135 (10)	[359 161]: 161 (100), 133 (20)	[36]
**11**	Rosmarinic acid isomer	17.44	251, 291, 329 max	359 (100)	[359]: 223 (25), 197 (30), 179 (50), 161 (100), 133 (5)	[359 161]: 161 (100), 133 (15)	[36]
**12**	Diosmetin-*O*-deoxyhexosyl-hexoside	18.18	252, 287, 335 max	607 (100)	[607]: 299 (100), 284 (20)	[607 299]: 299 (90), 284 (100)	[37]
**13**	Salvianolic acid E isomer	18.64	251, 284, 336 max	717 (100)	[717]: 519 (100)	[717 519]: 475 (10), 357 (100)	[31,38,39,40]
**14**	Salvianolic acid E isomer	19.34	251, 283, 331 max	717 (100)	[717]: 519 (100)	[717 519]: 475 (3), 357 (100)	[31,38,39,40]
**15**	Salvianolic acid B	20.95	252, 283, 313 sh	717 (100)	[717]: 537 (3), 519 (100)	[717 519]: 357 (15), 339 (30), 321 (100)	[31]
**16**	Salvianolic acid A isomer	24.86	288, 300, 320 max	493 (100)	[493]: 359 (100)	[493 359]: 223 (30), 197 (31), 179 (35), 161 (100)	[41,42]
**17**	Salvianolic acid A isomer	25.44	288, 300, 320 max	493 (100)	[493]: 359 (100)	[493 359]: 223 (20), 197 (25), 179 (30), 161 (100)	[41,42]
**18**	Isosalvianolic acid B	29.23	286, 300, 322 max	717 (100)	[717]: 519 (100)	[717 519]: 501 (2), 339 (100)	[38,43]
**19**	Salvianolic acid L	29.23	286, 300, 322 max	717 (100)	[717]: 519 (100)	[717 519]: 501 (2), 339 (100)	[38,43]

**Table 2:** Antioxidant activity of the infusion from *T. capitata* by DPPH and ABTS assays.

Method	IC_50_ (μg/mL) ^a^	TE (μM/μg Extract) ^b^
DPPH	19.85 ± 1.08	4.22 ± 0.31
ABTS	11.53 ± 0.27	1.05 ± 0.11

^a^ Mean ± SD, three independent experiments performed in duplicate. ^b^ Trolox equivalent.

## Data Availability

Data is contained within the article.
